# Identifying and Addressing Barriers to Uptake of Voluntary Medical Male Circumcision in Nyanza, Kenya among Men 18–35: A Qualitative Study

**DOI:** 10.1371/journal.pone.0098221

**Published:** 2014-06-05

**Authors:** Emily Evens, Michele Lanham, Catherine Hart, Mores Loolpapit, Isaac Oguma, Walter Obiero

**Affiliations:** 1 Health Services Research, FHI 360, Durham, North Carolina, United States of America; 2 Social and Behavioral Health Sciences, FHI 360, Durham, North Carolina, United States of America; 3 Amref Health Africa, APHIA*plus* IMARISHA, Nairobi, Kenya; 4 FHI 360, Kisumu, Kenya; 5 Health Policy and Administration, School of Public Health, University of Illinois at Chicago, Chicago, Illinois, United States of America and Nyanza Reproductive Health Society, Kisumu, Kenya; Karolinska Institute, Sweden

## Abstract

**Background:**

Uptake of VMMC among adult men has been lower than desired in Nyanza, Kenya. Previous research has identified several barriers to uptake but qualitative exploration of barriers is limited and evidence-informed interventions have not been fully developed. This study was conducted in 2012 to 1) increase understanding of barriers to VMMC and 2) to inform VMMC rollout through the identification of evidence-informed interventions among adult men at high risk of HIV in Nyanza Province, Kenya.

**Methods:**

Focus groups (n = 8) and interviews were conducted with circumcised (n = 8) and uncircumcised men (n = 14) from the two districts in Nyanza, Kenya. Additional interviews were conducted with female partners (n = 20), health providers (n = 12), community leaders (n = 12) and employers (n = 12). Interview and focus group guides included questions about individual, interpersonal and societal barriers to VMMC uptake and ways to overcome them. Inductive thematic coding and analysis were conducted through a standard iterative process.

**Results:**

Two primary concerns with VMMC emerged 1) financial issues including missing work, losing income during the procedure and healing and family survival during the recovery period and 2) fear of pain during and after the procedure. Key interventions to address financial concerns included: a food or cash transfer, education on saving and employer-based benefits. Interventions to address concerns about pain included refining the content of demand creation and counseling messages about pain and improving the ways these messages are delivered.

**Conclusions:**

Men need accurate and detailed information on what to expect during and after VMMC regarding both pain and time away from work. This information should be incorporated into demand creation activities for men considering circumcision. Media content should frankly and correctly address these concerns. Study findings support scale up and/or further improvement of these ongoing educational programs and specifically targeting the demand creation period.

## Introduction

Three randomized controlled trials conducted in Uganda, Kenya and South Africa between 2002 and 2006 demonstrated the efficacy of voluntary medical male circumcision (VMMC) to partially protect men against HIV infection acquired through vaginal sex [1]–[4]. In these trials, infection rates in medically circumcised men were 50–60% lower than those among the uncircumcised group and VMMC services are now being scaled-up in 14 sub-Saharan countries, including Kenya [5].

As with the other 13 sub-Saharan countries, Kenya is experiencing a generalized HIV epidemic, with an overall prevalence of 6.4% among adults [6]. According to the 2008–2009 Kenya Demographic and Health Survey, the largest increase in HIV prevalence among men occurs between ages 20–29 with prevalence peaking at 10.4% among men 35–39, indicating the importance of targeting adult men with HIV prevention efforts [6]. The area formerly known as Nyanza Province has both the highest HIV prevalence (13.9%) and the lowest prevalence of male circumcision (44.8%) in Kenya [6].

In November 2008, the Government of Kenya (GoK) launched a National Program of Voluntary Medical Male Circumcision with a goal of circumcising 1.1 million men ages 15–49 years old across the country by 2013 [7]. In the Nyanza region, the target is to increase the proportion of men circumcised from 47% to 80%–or to perform 426,500 circumcisions over a four-year period. Kenya has had notable success with scale up, with male circumcision prevalence in Nyanza increasing from 48% in 2007 to 66% in 2012 [8]. From 2010 to 2012, Kenya’s Rapid Results Initiatives (RRIs), the periodic, intensive programmatic efforts to provide VMMC services, increased the numbers of males coming for VMMC, with particular success among adolescents; however, the number of adult men who have been circumcised remains lower than desired [5], [6], [9], [10]. Given this age group’s high risk of acquiring HIV, increasing VMMC uptake in this age group is likely to have the most immediate impact on population-level HIV incidence in Nyanza. In order to increase uptake, the identification of barriers to seeking VMMC and evidence-informed interventions to address those barriers is needed [6], [10], [11].

Previous research identified barriers to VMMC uptake at individual, health systems and socio-cultural levels; though these were largely identified through quantitative research methodologies with closed-ended questions. The most consistent barriers to uptake at the individual level included: cost (including financial and opportunity costs such as lost income due to time away from work) [9], [12]–[14], fear of pain [12]–[16], concern about safety during surgery and adverse events following the procedure [9], [12], [13], [16], and the post-procedure abstinence and healing period [16]–[18]. Health systems barriers include: transport costs, inadequate clinic facilities, lack of equipment and supplies, insufficient number of trained providers, and lack of experience regarding how and when to provide fixed versus mobile services [9], [19].

Finally, socio-cultural barriers, including cultural norms, ethnic identity and religious affiliation have been identified as factors central to the acceptance and uptake of VMMC [13], [16]–[18], [20]. Barriers at the individual and societal levels are of particular concern for men over 18 who are more likely to be in sexual relationships, hold jobs, and be active members within their ethnic and religious communities.

The lower-than-desired uptake of VMMC among adult men is motivating the GoK and implementing partners to develop new strategies to increase the numbers of men seeking VMMC services. National and provincial VMMC task forces and implementing partners are piloting new approaches to recruit men over the age of 25 for services [5], [9], but while the need for programmes to target older men has been identified, evidence-based strategies to accomplish this have not been fully developed or tested [11]. Additionally, input from key groups such as female partners, employers, and community and religious leaders, who may influence men’s perceptions of barriers to VMMC uptake and their ability to overcome these barriers, has not been widely gathered.

The goal of this research was to 1) increase understanding of the factors that serve as primary barriers to VMMC uptake in Nyanza Province among men at high risk of HIV, and 2) to inform VMMC rollout through the identification of evidence-informed interventions to increase uptake among adult men at high risk of HIV in Nyanza Province, Kenya.

## Methods

This study was conducted in 2011–2012 in the urban Kisumu East and rural Rachuonyo districts of Nyanza Province; prior to 2013 provinces were used as the primary administrative unit, after the 2013 shift countries became the primary unit. All categories of participants were recruited so that they were evenly split between these two districts. Participants were purposively recruited to meet quota targets sufficient to reach data saturation [21]. Data collectors were male and female community members, fluent in both English and Dholuo, the primary local languages. All data collectors had previous experience conducting qualitative research and received additional training in research ethics and qualitative research methods by the research team leaders. Community mobilizers and groups who were engaged in efforts to encourage men to adopt VMMC assisted with recruitment by identifying potential study participants during outreach efforts. Male and female participants referred partners who they thought might be interested in participating in the study. While male participants included members of the general male population, there were specific efforts to recruit men in high-risk occupational groups. Local VMMC implementing partners have identified selected occupational groups including fishermen and transport industry workers (boda-boda riders, tuk tuktuk and matatu drivers, touts) and jua kali artisans (a Swahili term that refers to the informal sector of the economy) as at high risk of HIV infection [22]–[24]. Leaders of high risk occupational groups helped community mobilizers and data collectors identify potential participants. As far as the authors are aware, previous research to identify barriers to uptake specific to these groups has not been conducted.

Focus group discussions (FGD) groups were conducted with men (n = 8, 95 participants total) and were stratified by age (18–24 and 25–35) and circumcision status (uncircumcised and medically circumcised within the last 6 months). Twenty-two in-depth interviews (IDIs) and were then conducted with medically circumcised (n = 9) and uncircumcised men (n = 13) ages 18–35. Men participating in the IDIs were not the same as those who participated in the FGDs.

IDIs were also conducted with female partners of circumcised (n = 8) and uncircumcised men (n = 12). Female partners of men in the above high-risk occupational groups were targeted during recruitment. Some, but not all, of the women interviewed were partners of male study participants.

Finally, IDIs were conducted with three other groups believed to have an influence on men’s circumcision decision-making: 1) healthcare workers providing VMMC (n = 12), 2) community and religious leaders (n = 12) and 3) employers and trade union representatives (n = 12). These participants were identified through relationships established by the research team during VMMC promotion activities and through referrals from other participants.

Participants selected their preferred language–English or Dholuo–for the interview. The focus group and interview guides included questions about individual, interpersonal, health systems and societal barriers to VMMC uptake and potential ways to overcome these barriers. All focus groups and interviews were conducted either in community settings convenient to participants or in a private office of a local NGO. Data collectors were instructed to conduct interviews and focus groups in settings where discussions could not be overhead; all interviews and focus groups were audio-recorded. On completion of each focus group, the note taker and moderator together used their handwritten notes and the audio-recording to produce expanded notes in English. Interviews were simultaneously translated and transcribed verbatim in English [25].

The research team leaders were US-based public health qualitative methods experts with previous research experience in Kenya. They reviewed transcripts as they were completed to ensure quality of interviews. After reviewing transcripts the team determined that data saturation had been reached. After the completion of data collection, a rapid analysis of the data was conducted to identify men’s primary barriers to VMMC in order to inform the provincial Ministry of Health’s annual program planning process. In February 2012, the study team organized and facilitated a VMMC stakeholder workshop in Kisumu. The workshop included representatives of the Ministries of Health, VMMC service providers, demand creation partners, and agencies funding VMMC efforts. The workshop presented preliminary study findings and engaged stakeholders in the identification and prioritization of the most feasible and acceptable interventions for addressing the main barriers to VMMC uptake.

Following the stakeholder workshop, the research team conducted rigorous coding and analysis of the data. Through a standard iterative process [26], they developed and used a codebook to structurally and thematically code the interviews in a qualitative data analysis software program, QSR NVivo version 9. For each category of interview or focus group, two data analysts independently coded transcripts, and inter-coder agreement was assessed [27]. This process was repeated until inter-coder agreement of at least 80% was reached for each category of interviews. After that, each transcript was coded by one analyst, although inter-coder agreement was assessed at regular intervals. The team updated the codebook after each inter-coder reliability check and as new themes emerged. Code reports were generated, and an inductive thematic analysis [28] was conducted to address the study objectives.

### Ethics Statement

The study was approved by the institutional review boards at FHI 360 and the Kenya Medical Research Institute (KEMRI) and all research was conducted in full compliance with the approved study protocol. Each participant provided verbal informed consent prior to study participation. Verbal consent was sought because: 1) the study was minimal risk 2) the data collection instruments did not require disclosure of identifying information and 3) a written signature would provide the only link between the participant and the study so verbal consent ensured greater confidentiality for participants. By approval of the above institutional review boards, data collectors documented participant consent by signing and dating the informed consent forms after obtaining verbal consent.

## Results

Men ranged in age from 18 to 35 with a mean age of 25.7; 47% were over the age of 25; and, 51% were married. Both uncircumcised and circumcised men identified the top barriers to VMMC uptake to be financial concerns and fear of pain during the procedure and recovery. These were also the primary concerns identified by women. However, the female partners of uncircumcised men expressed few concerns overall.

### Perceived Pain

General fear of pain regarding circumcision was the concern mentioned most often. Men expressed concern over pain specifically during the VMMC procedure but they also feared pain during recovery due to potentially poor suturing or a surgical mishap resulting in a deformity. A few men thought that pain could result from a lack of follow-up wound assessment by VMMC health providers. Concern over pain during morning erections was another common concern. Some men and women feared that pain during recovery would cause a man to miss work and lose income.

*“I was afraid because those who had gone for the circumcision were saying that it was very painful when the foreskin is cut, during suturing, and then after that you were unable to perform your normal duties as usual. Being a person with dependents, it was not appealing to me.”*

- Circumcised man, 24 years old, Rachuonyo

However, concern about pain was not universal; eight men–four of whom were not circumcised at the time of their interviews–said that they were not concerned about pain.

*“No, no, right now I am 30, and there is no pain I will feel when I become circumcised because I have endured more painful things so there is none I will feel when I become circumcised.”*

- Uncircumcised man, 30 years old, Kisumu East

Circumcised men and women with circumcised partners were asked about pain during the post-circumcision recovery period. All of the circumcised men reported that they managed their pain well during recovery by following the post-circumcision instructions given to them by their VMMC providers–primarily, taking prescribed painkillers and urinating when waking with a morning erection. They said their circumcision experiences were not as painful as they feared prior to the procedure.

*“The provider who performed the circumcision gave me some pain killers, and I used them as prescribed to me. The drugs really helped me such that the pain was bearable…I did not have too much pain.”*

-Circumcised man, 24 years old, Rachuonyo

### Financial Concerns

Financial concerns included concern about missing work and losing income during the procedure and healing, potentially losing one’s job from being absent, and how one’s family would survive if the father was out of work during the recovery period. This last concern was mentioned only by men. Some men described difficulty in taking even a short break away from work for fear that they would lose their jobs.

*“Since I have children and a wife…I’m the one taking care of them, I’m the daily breadwinner, so it will be very difficult to survive because…I’ll take some time before I go back to job.”*

- Uncircumcised man, 23 years old, Kisumu East

*“It will be difficult for my people to eat because I will be forced to stay in the house since I cannot go to work as I would still be healing.”*

- Uncircumcised man, 25 years old, Rachuonyo

These concerns were greatest among uncircumcised men and women with uncircumcised partners. Men between the ages of 25 and 35 expressed more concern than younger men. Slightly more than half of all women stated that men’s financial concerns were not significant or could be overcome with their assistance; of these women, most had uncircumcised partners.

The majority of circumcised men reported returning to work soon after the procedure–either immediately following VMMC or within two days after the procedure. Two men took a considerable amount of time off, reporting they took a month away from work. More than half of the circumcised men stated that VMMC did not affect their earning capacity.

*“I did not miss work, I worked before and after circumcision, for they had told us that it could be done and you just do your normal duty. Before they talked to me it was a worry how would I eat but when I found out that it could be performed and report to my duties immediately then it was not a concern, I came back and worked.”*

- Circumcised man, 27 years old, Kisumu East

Female partners of circumcised men reported their male partners were out of work much longer than reported by the male participants. Only one woman reported that her partner returned to work shortly after VMMC. Five women reported their partners were away from work between one and four weeks and two women reported their partners were away from work for more than one month. Despite this, women did not report significant financial stress resulting from the time their partners were not working; many reported they supported their families following circumcision by taking over their husband’s responsibilities or working more at their usual job while their partners were away from work.

**Interviewer: Were there financial effects on your household?**

*Respondent: No, there was no financial constrain.*

**Interviewer: Why are you saying that?**

*Respondent: It is because I am the one who was doing the work that he was supposed to be doing to sustain us as he was still healing.*

- Female partner of a circumcised man, 19 years old, Rachuonyo

Occupation also appears to be a factor in men’s hesitation to seek VMMC services. Men working in the transport sector (for example, boda-boda drivers) and those engaged in jobs involving manual labor or being in the water (fishermen) said they were reluctant to seek VMMC services due to the physical demands of their job.

Finally, some men’s financial concerns seemed to stem from misinformation. Some men confused the period they would need to be away from work (1–3 days is recommended) following VMMC, with the prescribed 6-week period of abstinence following VMMC, while others were unclear on the amount of time they would be away from work. Three women also had misconceptions about the time men would need to be away from work–ranging from 2 weeks to 6 months.

*“The six weeks, that is about two months, and if your ways of getting a living is stopped for two months and you have a family that needs to feed then you will find that you have developed a difficult gap.”*

- Uncircumcised man, 35 years old, Kisumu East

### Secondary Barriers

IDIs and FGDs with men also explored a number of other potential barriers to VMMC, including the abstinence period, voluntary counseling and testing as part of VMMC services, female partners’ opinions of circumcision, sexual function after VMMC, potential adverse events, cultural concerns, and access to and quality of VMMC services. These concerns were mentioned significantly less often than the commonly cited concerns with perceived pain and financial issues.

The most common secondary barrier was the post-procedure abstinence period. Attitudes towards the abstinence period were mixed with approximately one-third of men in IDIs and more than half of FGDs citing abstinence as a concern, while nearly half of men in IDIs stating it was not problematic. There was no major difference in response by circumcision status or age group. For men who were concerned about the abstinence period, their primary concerns were that it would be difficult to deal with the urge to have sex during the abstinence period, that it was a long time to abstain, and that their female partner would have issues with the abstinence period. Men who were not concerned with the abstinence period felt that it was not too long to abstain, that it was worth the long term benefits, and that so long as their female partner knew why they were abstaining, she would understand. Several men recommended talking with partners about the abstinence period. Concerns over culture, adverse events and sexual function were few. VCT, female partners’ opinion, and access and quality of services were not found to be major barriers to uptake [29].

### Interventions to Increase Uptake of Circumcision

Data on interventions to address the primary barriers to uptake of VMMC came from men, women, health providers, employers and community and religious leaders. Additionally, participants at the stakeholder workshop developed and prioritized interventions to address uptake of VMMC. All of the following interventions were both prioritized by study participants and endorsed by workshop attendees.

### Interventions to Address Concerns about Perceived Pain

Interventions to address concerns over pain focusing on two main themes: 1) improving messaging regarding expected pain and pain management, and 2) improving the mechanisms for delivering those messages.

**Adequate and detailed messages** about the procedure and pain management were the most commonly mentioned way to address fear of pain. Participants said that these messages should: provided information about pain management before and after the procedure; correct misinformation and educate men on the differences between traditional circumcision–where anesthesia is not provided–and VMMC by health providers. Participants also recommended providing motivational messages focused on overcoming fear and helping men understand the long-term benefits of circumcision compared to the short and manageable pain from VMMC.

*“Before I went for circumcision, I used to think that circumcision brings a lot of pain, and the pain would take long especially on my penis, but the person who advised me told me that when you go for circumcision, … on that very day there is a drug that they use and you will not feel pain until after three hours you will feel a little but it’s pain that is bearable and you can go about your duties. That’s why I had the courage to go.”*

- Circumcised man, 23 years old, Kisumu East

**Information on healing**, particularly adequate instructions–both oral and written–on wound care was mentioned by health providers, men, and women as important for curbing men’s concerns around pain following VMMC. Providers also stated that including female partners would promote proper healing through improved wound care at home.

*“They told me that…it would heal if you follow their instructions…they also give you a phone number and in case you have a problem you may call for assistance.”*

- Circumcised man, 28 years old, Rachuonyo

Study participants, especially the men themselves, and workshop participants recommended **using circumcised peers** to address men’s fear of pain. Positive peer testimonials were discussed as having a potentially strong influence on men and men also stated they were more likely to seek VMMC services when they saw other men healing well after the procedure.

*“In addressing fear of pain, those who have undergone circumcision should be used as ‘lesson objects’. We can use real people and practical names to create awareness among others by sharing their own experiences about the procedure.”*

*-* Community leader, Kisumu East

Finally, workshop stakeholders also suggested expanding or improving VMMC media messages to educate the community, especially targeting men between the ages of 18 and 35, on pain and promote the benefits of circumcision.

### Interventions to Address Financial Concerns: Provision of Money and Food

The most highly prioritized intervention to address financial concerns among men was the **provision of money** following VMMC–to compensate for lost wages and/or provide for family needs such as food, rent or children’s school fees. This was mentioned more often by uncircumcised men, men between the ages of 25 and 35, and women with circumcised partners. Several women also suggested that women could provide support for their families either by working more or taking over their partner’s job responsibilities following VMMC; this was not mentioned by men.

*“If there is something that can be done that can make me go for circumcision, the problem of income should be addressed because after circumcision you need to relax for a few days, if…my daily income can be squared, if it can be worked out so that when I’m down my family can survive for some time, I can go for circumcision.”*

- Uncircumcised man, 25 years old, Kisumu East

While of the majority of providers and community leaders supported this intervention, a number of providers and community leaders felt that providing cash was neither necessary nor feasible. Community leaders expressed the belief that men do not need financial support after circumcision, either because the actual need for assistance was low, because men would not want to take money from others or because they would not want their decisions around VMMC to be public knowledge. A related intervention–the **provision of food or food vouchers** to men following VMMC–was also discussed, though less frequently, and more often by uncircumcised men. A small number of women, community leaders, and health providers also supported this option.

Stakeholders felt that providing money or food vouchers to men or their female partners could compensate men for lost income while alleviating financial concerns and providing for family wellbeing. They also suggested that partnering with a supermarket to provide non-taxable items could directly address the concern of feeding one’s family while alleviating their concern that men might use cash for unintended purposes.

### Interventions to Address Financial Concerns: Using Savings and Providing Education on Saving

Another commonly mentioned intervention to address financial concerns was **using savings** to cover expenses during healing or **providing men with education on how to save** in advance of the procedure. A few participants stated that saving was possible while some also stated it would not be possible for some men given their limited incomes.

*The [savings] program will be a good idea, because maybe he does not know how to save his money. Some people are really spendthrifts, they use their income to the last cent, so in case an emergency comes up like disease; they don’t have money to use to buy drugs or food. This will make one to have stress of feeding the family. So it would be better if he saves money for future use.*

- Female partner of a circumcised man, 19 years old, Rachuonyo

### Interventions to Address Financial Concerns: Employer Benefits

Employer benefits–including an allowance of time off or a change of duties following VMMC–were identified as a potential intervention by both men and women with circumcised men mentioning them slightly more often than uncircumcised. Many employers and trade union representatives reported providing or planning to provide benefits to employees who sought VMMC and several others discussed willingness or plans to provide paid leave or time off following VMMC. However, one-third of employers stated they were not financially able to provide these benefits because of limited resources.

**Interviewer: So you cannot also pay for the time they would be off work while healing?**

*Respondent: No, what I can do is may be to accept them back to work once they have healed from the surgery.*

- Owner of a carpentry workshop and food joint, Rachuonyo district

### Interventions to Address Financial Concerns: Educating Men on Anticipated Time Away from Work

Finally, circumcised men, and a small number of women with uncircumcised partners, recommended providing counseling or education to men on the actual time they would be away from work. Health providers and community leaders mentioned counseling and education more often as a means of lessening financial concerns and clarifying misconceptions about the financial burden of circumcision. During the stakeholders’ workshop, an intervention combining education on expected time away from work with efforts to target services to times when certain occupational groups are less likely to be working such as providing services for fishermen during the months when fishing is prohibited or on non-market days for transport workers.

## Discussion & Conclusions

This study was designed to identify why adult men at high risk of HIV infection are not seeking VMMC services in the numbers needed to have a rapid impact on the HIV epidemic in two districts of the former Nyanza Province, Kenya, and to identify potential interventions to address these obstacles. Previous research has been limited to using mostly quantitative methods with closed-ended questioning to investigate barriers to circumcision. We used qualitative exploration as a broader means to enhance our understanding of barriers and identify strategies to increase uptake. Additionally, we investigated the role that influential others play in men’s decision to seek, or not to seek, circumcision services. Understanding social, cultural, physical and economic factors that influence men’s decisions to seek VMMC services can inform the development of successful interventions to address barriers and capitalize on facilitating factors.

Two primary concerns with VMMC emerged from this analysis: financial concerns and fear of pain during and after the procedure. Men between the ages of 25 and 35 expressed particular concern for the financial implications of seeking VMMC. This was not surprising as these older men are more likely to be married and have families to support resulting in a more significant negative economic impact due to missed work. Concern about the financial burden of VMMC was especially common among men who earned a daily wage such as fishermen and those in the transport sector. While the focus on high-risk groups, including daily laborers, is a potential limitation of this study, this group is of significantly represented in Nyanza which has unemployment rates of approximately 72% [6]. However, it is important to note that study results, especially those related to financial concerns, may not be generalizable to men who earn regular salaries or work in office environments.

Study participants and workshop stakeholders were aligned on key interventions to address men’s financial concerns. A food or cash transfer to compensate men for time away from work and ensure men’s families were provided for during the healing period emerged as the highest priority intervention. Circumcised men reported being away from work a much shorter time than was reported by their female partners. This discrepancy is noteworthy and deserves additional exploration. Regardless of the reason for the differences between men’s and women’s reports, a small financial compensation for lost wages during the time men are away from work could motivate them to seek VMMC; a randomized controlled trial investigating the effect of food vouchers on the uptake of VMMC for men between the ages of 25–49 has recently been conducted in Nyanza.

Two other interventions recommended to address men’s financial concerns, education on saving in and employer-based benefits could be implemented at a fairly low cost; however, their potential impact is likely limited. Many men with small daily incomes–a large group of those targeted for VMMC–may be unable or unwilling to save for the procedure. Similarly, employer-based benefits have the potential to be important for those men in formal employment however, the majority of men in Nyanza are self-employed or do not work for an employer of sufficient size to be able and willing to implement this approach.

It is important to highlight that not all men expressed financial concerns about VMMC; a sizable number of men reported that missing work or losing income was not a barrier to circumcision. Additionally, more than half of women stated that financial concerns were not significant or could be overcome with their assistance. This variation suggests potential differences in the financial situations of participants, differing viewpoints by gender, or discrepancies between the expectation of financial hardships following VMMC and the reality of the experience. Educating men on what to expect following VMMC could be an important tool for increasing uptake among men 18–35. Given the low number of days circumcised men reported being away from work as well as the minimal negative financial impact reported by women, peer testimonials and improved counseling messages could help men overcome their financial concerns and would be relatively easy to incorporate into existing demand creation and service provision activities. Education on what to expect and improved wound-care instructions following VMMC could also alleviate confusion between the recommended six weeks of abstinence following VMMC and expected time away from work.

Interventions to address men’s concerns about pain were organized around two themes: refining the content of counseling and educational messages about pain and improving the ways these messages are delivered. Counseling on pain was identified by both community leaders and providers as a good approach to educate men on the benefits and realities of recovery from VMMC. Updating counseling and training materials for health providers and community mobilizers to include detailed and accurate information on pain management and expectations could be a relatively low-cost intervention to address these specific concerns. Along with testimonials from circumcised peers, messages that cultivate a sense of courage in the face of potential pain could also help address fears.

New methods of circumcision recently studied in Kenya, Rwanda, and Zambia, using nonsurgical VMMC methods such as the Shang Ring and PrePex devices, rather than the standard surgical method, may help address some concerns about pain and time away from work. While additional acceptability data on new devices is needed, these new methods are minimally invasive, take less time to perform than standard surgical methods (6–7 minutes vs. 20–40 minutes) and do not require suturing. In these studies, no severe adverse events were reported which were attributable to the devices and mild to moderate adverse events, including the reports of pain, were minimal [30]–[32].

Ultimately, men need accurate and detailed information and counseling on what to expect during and after VMMC regarding both pain and time away from work. This information should be incorporated into demand creation activities undertaken by community mobilizers and peer educators for those men considering circumcision. Counseling and education from VMMC providers should also include these messages and media content could frankly and correctly address these concerns. The provision of demand creation and educational messages specifically addressing men’s concerns about pain and lost work time is the strongest recommendation to emerge from this research. We acknowledge that educational programs being implemented in Nyanza are likely already delivering such messages; these study findings support scale up and/or further improvement of these ongoing educational programs and specifically targeting the demand creation period.

These results reflect men’s concerns with VMMC after a considerable amount of programmatic effort promoting VMMC within Nyanza. Religious and cultural barriers including tribal, community, family and peer-based concerns as well as concerns over sexual functioning following circumcision which were found to be barriers in earlier acceptability studies, were not identified by men and women as major concerns in this study. Religion and culture no longer being major barriers to VMMC uptake highlights the shift in social norms over the life of Kenya’s VMMC program and illustrates the acceptance of VMMC in Kenya–and specifically among the Luo. While the authors are unaware of any published data documenting men’s attitudes on sexual functioning post-circumcision, anecdotal evidence indicates unchanged or improved sexual functioning after the procedure. As the numbers of medically circumcised men increases, peer communication highlighting the lack of negative impact on sexual experience seems to have helped address men’s concerns in this regard.

It is also noteworthy that access to and quality of VMMC services did not emerge as barriers to VMMC uptake. This demonstrates the triumph of the GoK’s efforts to promote VMMC and highlights the importance of ensuring accessible, high-quality service. Service access and quality continue to be paramount for ensuring that safe procedures are available to men who will benefit most. Ongoing support to the GoK will be essential to ensure that evidence-driven improvements to services advance the important gains to date.

This study confirms previous research identifying perceptions of pain and financial concerns as key barriers to VMMC and provides further insight into reasons why VMMC uptake among adult men remains lower then desired. Confirmation of these barriers illustrates the challenge of addressing these concerns and reinforced the importance of developing interventions to address them. Additionally, this study provides intervention ideas generated and prioritized by the target VMMC population, influencing individuals or groups, and the various stakeholders working in Nyanza to increase VMMC uptake among older men in Kenya. There results could be adapted for other traditionally non-circumcising communities in sub-Saharan Africa. Results should be also be considered for VMMC programs using new devices. As VMMC strategies and programs are designed and scaled-up globally, interventions to target barriers to circumcision are essential for generating demand among older men, a group essential for ensuring the ability of VMMC to reduce HIV incidence by 2015.
